# Supplementary material for: Population imaging at subcellular resolution supports specific and local inhibition by granule cells in the olfactory bulb
Source: Sci Rep. 2016 Jul 8;6:29308. doi: 10.1038/srep29308 (PMC4937346; doi:10.1038/srep29308)
Supplement: Supplementary Figures [file srep29308-s1.pdf]

Population imaging at subcellular resolution supports specific and local inhibition by granule cells in the olfactory bulb

Martin Wienisch and Venkatesh N. Murthy

Supplementary Figures

Supplementary Fig. S1: Viral labeling of granule cells with dTomato and GCaMP3. **A**. Tissue sections from OB of mice injected with AAV2/1 virus carrying genes for GCaMP3 and dTomato (insert sequence schematized on top). Red and green channels are native fluorescence from the two proteins, and DAPI signals (blue) highlight nuclei of cells. Note dense signal in the external plexiform layer (EPL), where the apical dendrites of GCs reside, and in the granule cell layer (GCL). Mitral cells are largely unlabeled and are seen as ghosts in the fluorescence images. **B**. Cre-dependent expression of dTomato and GCaMP3 in a VGAT-Cre mouse. Tissue sections show excellent colocalization of dTomato and GABA, with no expression in GABA-negative mitral cells. **C**. OBs from living mice imaged (epifluorescence) through a cranial window after viral expression of dTomato and GCaMP3 (top). **D**. Labeled GCs imaged (dTomato channel) in vivo using two-photon microscopy. Optical sections at different depths reveal dendrites, spines and cell bodies. Expression is sparse in this example because of low volume of virus injection.

Supplementary Fig. S2: Control experiments confirm reliability and rule out some biases. **A**. Scatter plot of odor-evoked fluorescence signals across two trials plotted for >2500 GC-odor pairs. A linear fit and the 95% confidence intervals are shown in red. The confidence interval is around 8% of the mean. **B**. The maximum response of each GC (across the 20 odors used) did not correlate with the resting fluorescence ($p = 0.134$). This suggests that resting fluorescence (for example, due to expression levels) is not the main determinant the magnitude of responses. **C**. Blocking inhibition with bicuculline reveals that most GCs can become activated. Each column represents the time course of fluorescence intensity in 150 GCs during odor stimulation with two odors (ethyl tiglate at left and 2-methoxypyrazine at right). GCs are rank ordered in

decreasing response amplitude under control conditions. Note that after bicuculline infusion into the OB, most of the GCs respond to ethyl tiglate. Therefore, the lack of responses of many of the labeled GCs under control conditions is not due to an inability of these neurons to increase their fluorescence – for example, due to nonspecific loss of the indicator's calcium reporting ability. **D**. The percent of non-responding GCs within the imaged region (calculated as described in Methods) was high for most odorants under control conditions (blue), but went down significantly for most odors after bicuculline infusion (red).

Supplementary Fig. S3: Imaging with GCaMP5 confirms basic findings with GCaMP3. **A**. Responses of GCs as measured with GCaMP5, which has better sensitivity than GCaMP3. The average responses to 20 odors for each GC was arranged in descending order, and the resulting "tuning curves" averaged across 150 GCs from 2 experiments (red). For comparison, a null distribution was calculated from the baseline fluorescence fluctuations (black). From this data, we estimated that a GC responded to 6 odors on average (compared to 3 estimated with GCaMP3). **B**. The overall population activity of GCs for a given odor, measured with GCaMP5, was also highly correlated with the fraction of glomeruli activated by that odor. This confirms the findings using GCaMP3.

Supplementary Fig. S4: Responses of GCs in awake mice were stronger, but the basic findings from anesthetized animals hold. **A**. Imaged regions from the same mouse under anesthesia and when the mouse was awake. Although the two regions are different, they are very close to each other. It was technically difficult to return to the same region for the two imaging conditions. **B**. Time course of responses 50 GCs to two different odorants under anesthetized and awake condtions. GCs were more responsive in awake animals. **C**. Average tuning curves of GCs to 20 odors estimated by rank ordering odor responses for individual GCs and averaging them under anesthetized (red) and awake (blue) conditions. GC responsiveness

to odors is clearly higher in awake mice. **D**. The strong correlation between the extent of glomerular activation and the overall population activity of GCs is preserved in awake mice. **E**. Average responses of 50 GCs to increasing concentration of an odorant (allyl tiglate). GCs are rank ordered by the decreasing responsivity to the highest concentration. F. The average responses of all cells (black, dashed line) shows gradual increase in responses with increasing concentration, which is also seen for GCs with the strongest responses to the highest concentration (blue). However, responses of 5 GCs in the middle ranks revealed non-monotonic behavior (red), just as we observed for recordings in anesthetized animals.

Supplementary Fig. S5: Alternate sparseness measures. **A**. The relation between the fraction of glomeruli activated by an odor and the population sparseness of GCs (calculated as described in Methods). This experiment was in VGAT-Cre mice. The correlation coefficient was 0.83 and $p < 0.01$. **B**. Lifetime sparseness (calculated as described in Methods) across a population of GCs. Some measures for GC selectivity relies on using thresholds to define responses, making those measures sensitive to noise and other factors. Lifetime sparseness does not use any threshold for defining a response, and indicates how selective an individual neuron is to the range of stimuli. In our definition a value of 1 indicates equal responses to all stimuli and a value close to 0 indicates a very sparse code. We found that the lifetime sparseness varied from cell to cell, with an average value of 0.24 +/- 0.18 (N = 5 experiments, 637 GCs).

Supplementary Fig. S6: Glomerular inputs scale lawfully with increasing concentration of odors. **A**. Responses of 45 glomeruli from 2 OMP-GCaMP3 mice to increasing concentrations of allyl tiglate. Glomeruli were rank-ordered in decreasing response amplitudes for the highest concentration (right most panel), and the glomerular identities were then matched for the rest of the concentrations. **B**. Relation between responses and odor concentration for 6 glomeruli. The

glomeruli were chosen to span a range of responses to the highest odor concentration (note different scales on the Y axis). **C**. Average response amplitudes for 3 glomeruli in the top, middle and bottom ranges of response amplitudes to the highest concentration. Note that all responses were monotonically increasing, unlike what was seen for GCs.

Supplementary Fig. S7: Distribution of average pairwise separation of GCs randomly sampled from all the cells in the imaged region. From a population of ~150 cells in an experiment, 3, 5 or 20 cells were randomly chosen and their average pairwise separation calculated. This randomly sampling was repeated for a total of 1000 simulations. Note that the distribution became wider when only a few cells were sampled, but they were always symmetrically spread around the mean value.

Supplementary Fig. S8: Responses in apical dendrites of GCs. **A**. Example showing apical dendritic responses with dense labeling of GABAergic neurons with GCaMP3. Individual GC dendrites cannot be discerned, but overall fluorescence changes represent responses from populations of GC dendrites. **B**. Time course of responses of 10 regions of interest within the imaged field of view for all 20 odors, along with the average traces, are shown. **C**. Integrated dendritic activity is strongly correlated with density of glomerular activation. **D**. Average activity in the EPL is also highly correlated with average somatic activity of GCs measured in the same experiment. Circles with dense interior are 4 concentrations of allyl tiglate.

Supplementary Movie 1: Imaging GCs labeled with dTomato (and GCaMP3, not visualized here) using multiphoton microscopy. A stack of 400 optical sections (1 μm steps) starting at ~40 μm from the surface going up to ~440 μm deep. Apical dendrites with spines appear in the early

part of the movie, with deeper sections revealing GC somata. Field of view is approximately 300 μm X 300 μm.

Supplementary Movie 2: Movie showing the response of GCs to ethyl tiglate. Initial frames show resting fluorescence in gray scale, followed by relative changes in fluorescence (ΔF) color-coded such that greater increases are represented by redder colors. Duration of odor presentation is shown by red dot at top right, and the time is indicated at bottom right with 0 s corresponding to onset of odor stimulus. The reddest colors correspond to around 25% change in fluorescence from rest. Field of view is 300 μm x 300 μm.

Supplementary Movie 3: Movie showing the response of GCs to ethyl tiglate in the presence of bicuculline applied to the surface of the olfactory bulb (see Methods). Initial frames show resting fluorescence in gray scale, followed by relative changes in fluorescence (ΔF) color-coded such that greater increases are represented by redder colors. Duration of odor presentation is shown by red dot at top right, and the time is indicated at bottom right with 0 s corresponding to onset of odor stimulus. The reddest colors correspond to around 25% change in fluorescence from rest. Field of view is 300 μm x 300 μm.

Supplementary Movie 4: Movie showing the response of apical dendrites of GCs to ethyl tiglate. Initial frames show resting fluorescence in gray scale, followed by relative changes in fluorescence (ΔF) color-coded such that greater increases are represented by redder colors. Duration of odor presentation is shown by red dot at top right, and the time is indicated at bottom right with 0 s corresponding to onset of odor stimulus. Note that most responses appear as small puncta because of the nature of optical sectioning. The reddest colors correspond to around 25% change in fluorescence from rest. Field of view is 150 μm x 150 μm.

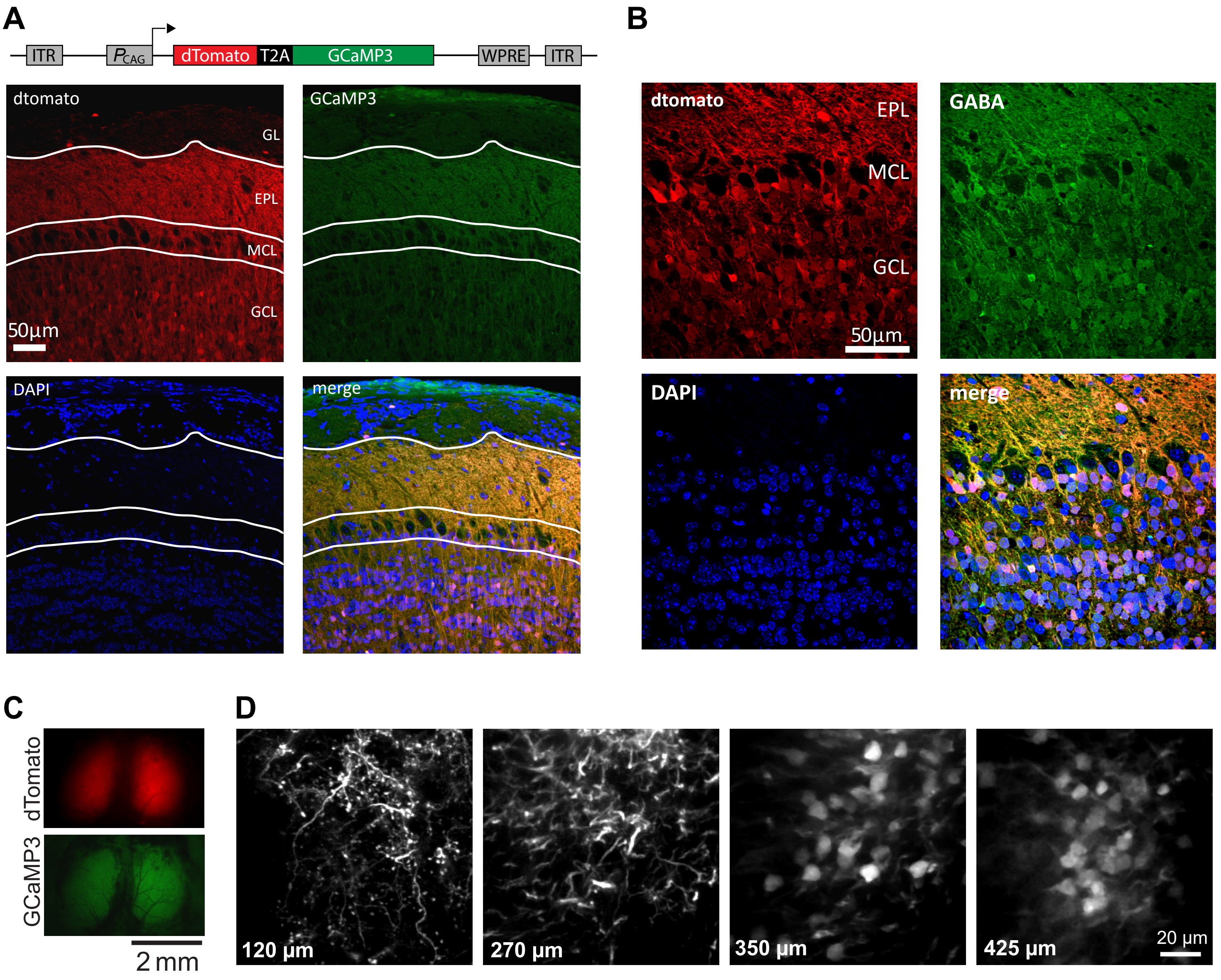
A
ITR
P_{CAG}
dTomato
T2A
GCaMP3
WPRE
ITR
dtomato
GL
EPL
MCL
GCL
50µm
GCaMP3
DAPI
merge
B
dtomato
EPL
MCL
GCL
50µm
GABA
DAPI
merge
C
dTomato
GCaMP3
2 mm
D
120 µm
270 µm
350 µm
425 µm
20 µm

Supplementary Figure 2

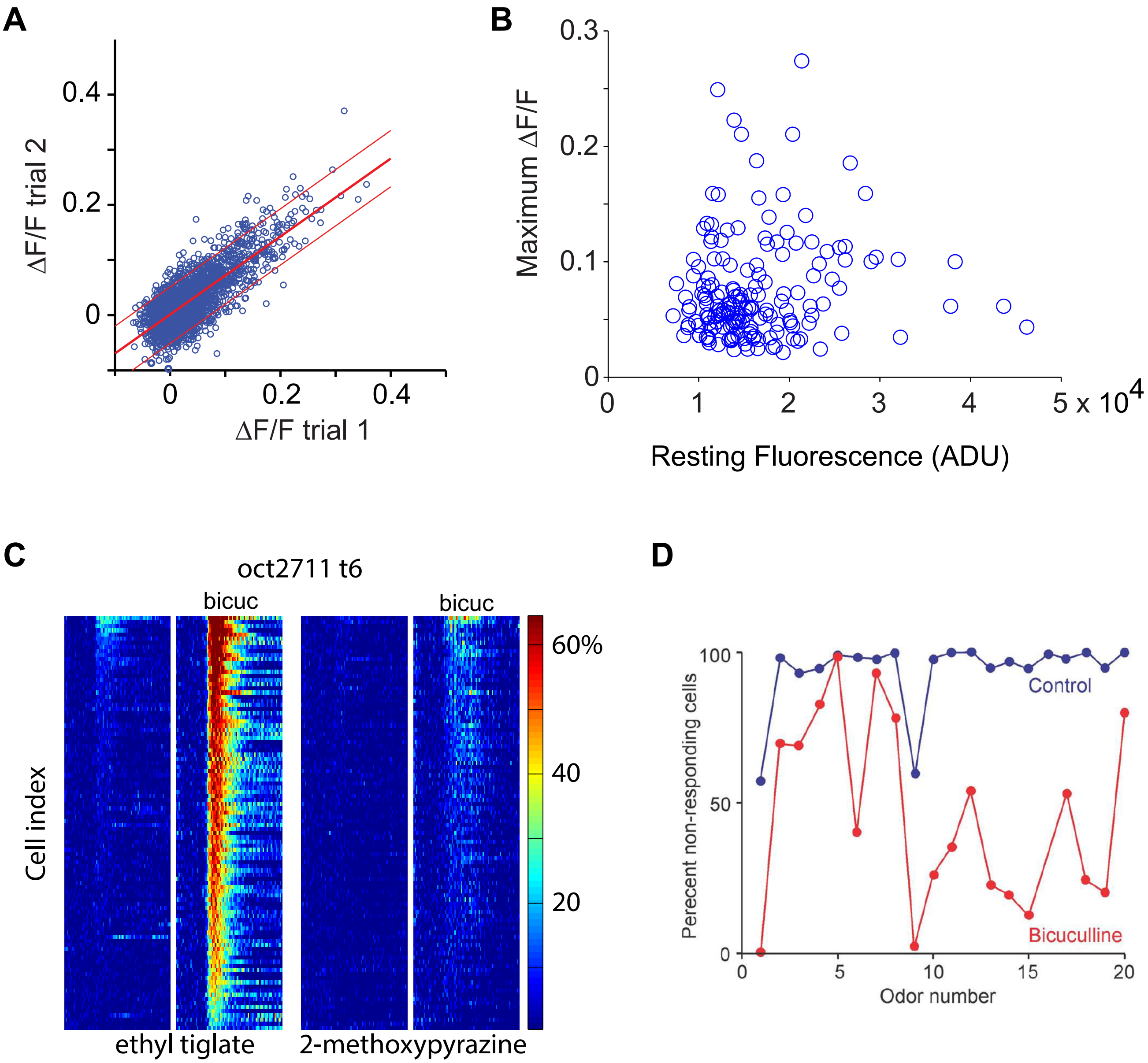

Supplementary Figure 3

A

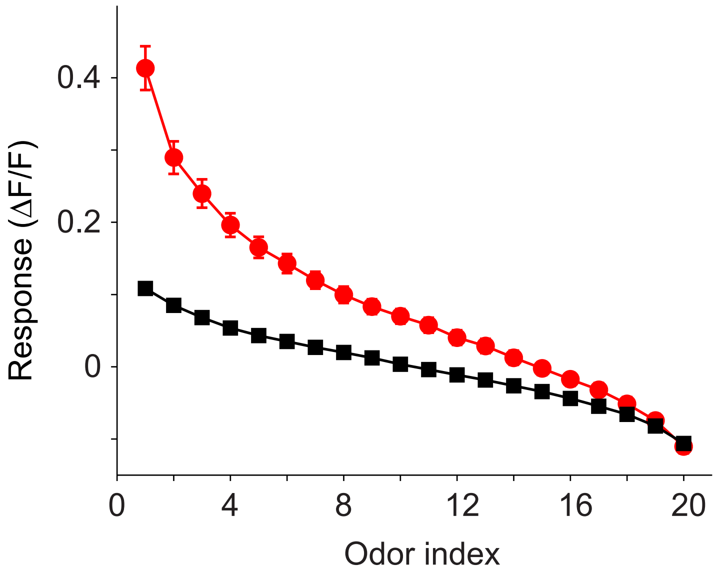

B

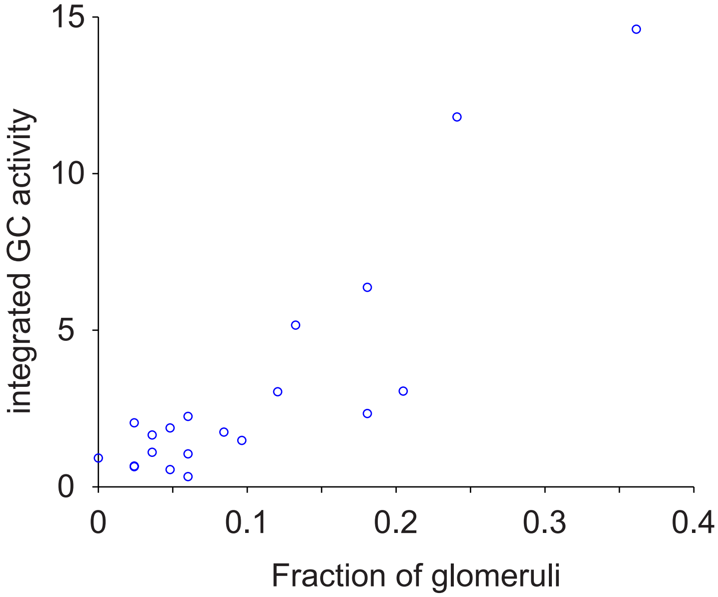

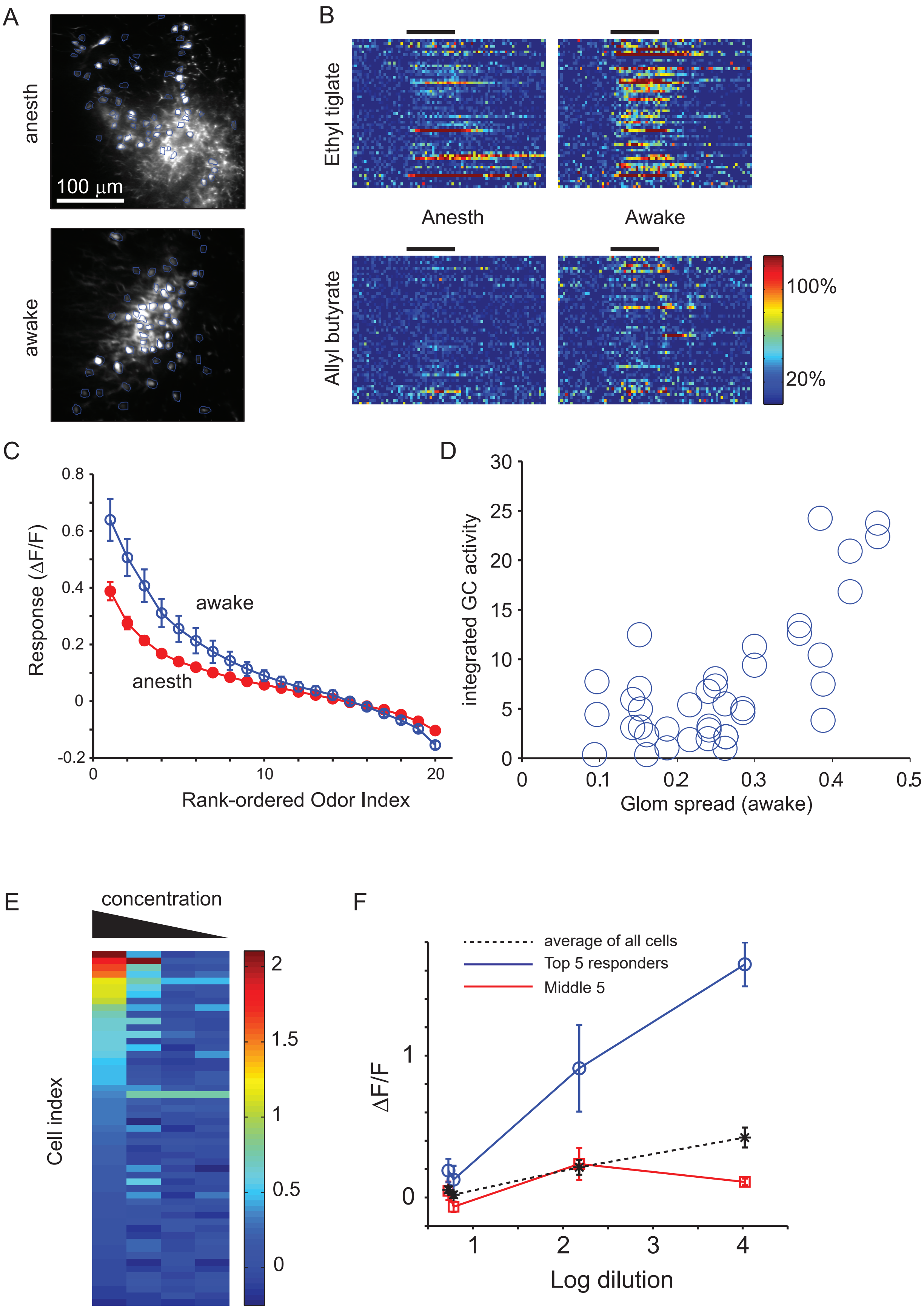

A
anesth
100 μm
awake
B
Ethyl tiglate
Anesth
Awake
Allyl butyrate
100%
20%
C
Response (ΔF/F)
0.8
0.6
0.4
0.2
0
-0.2
awake
anesth
0
10
20
Rank-ordered Odor Index
D
integrated GC activity
30
25
20
15
10
5
0
0.1
0.2
0.3
0.4
0.5
Glom spread (awake)
E
concentration
Cell index
2
1.5
1
0.5
0
F
average of all cells
Top 5 responders
Middle 5
ΔF/F
1
0
1
2
3
4
Log dilution

Supplementary Figure 5

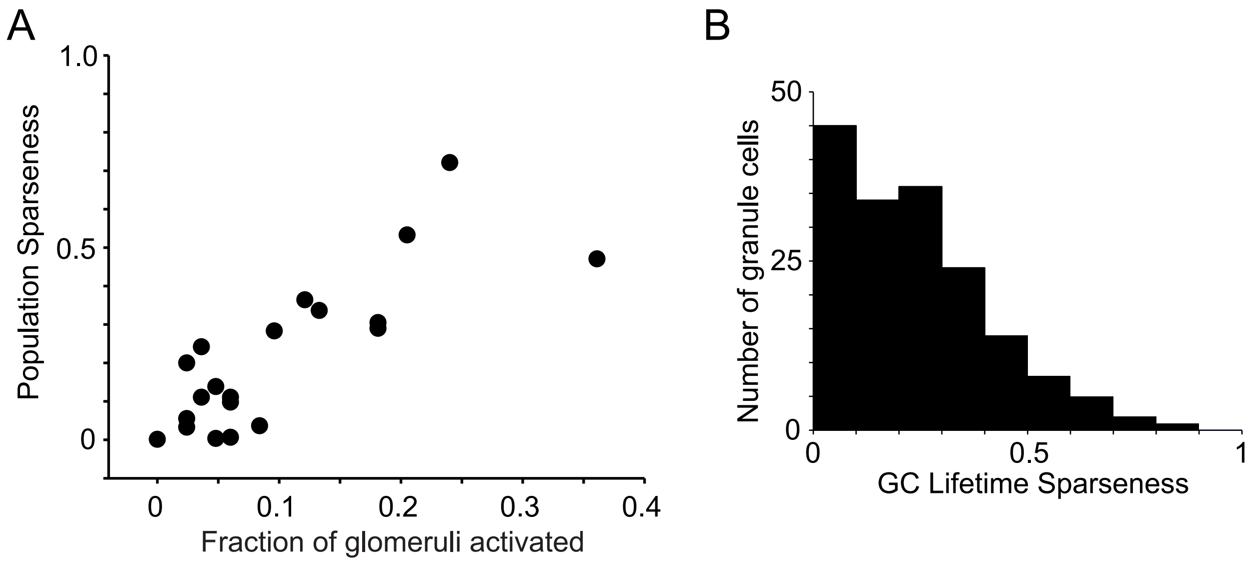

Supplementary Figure 6

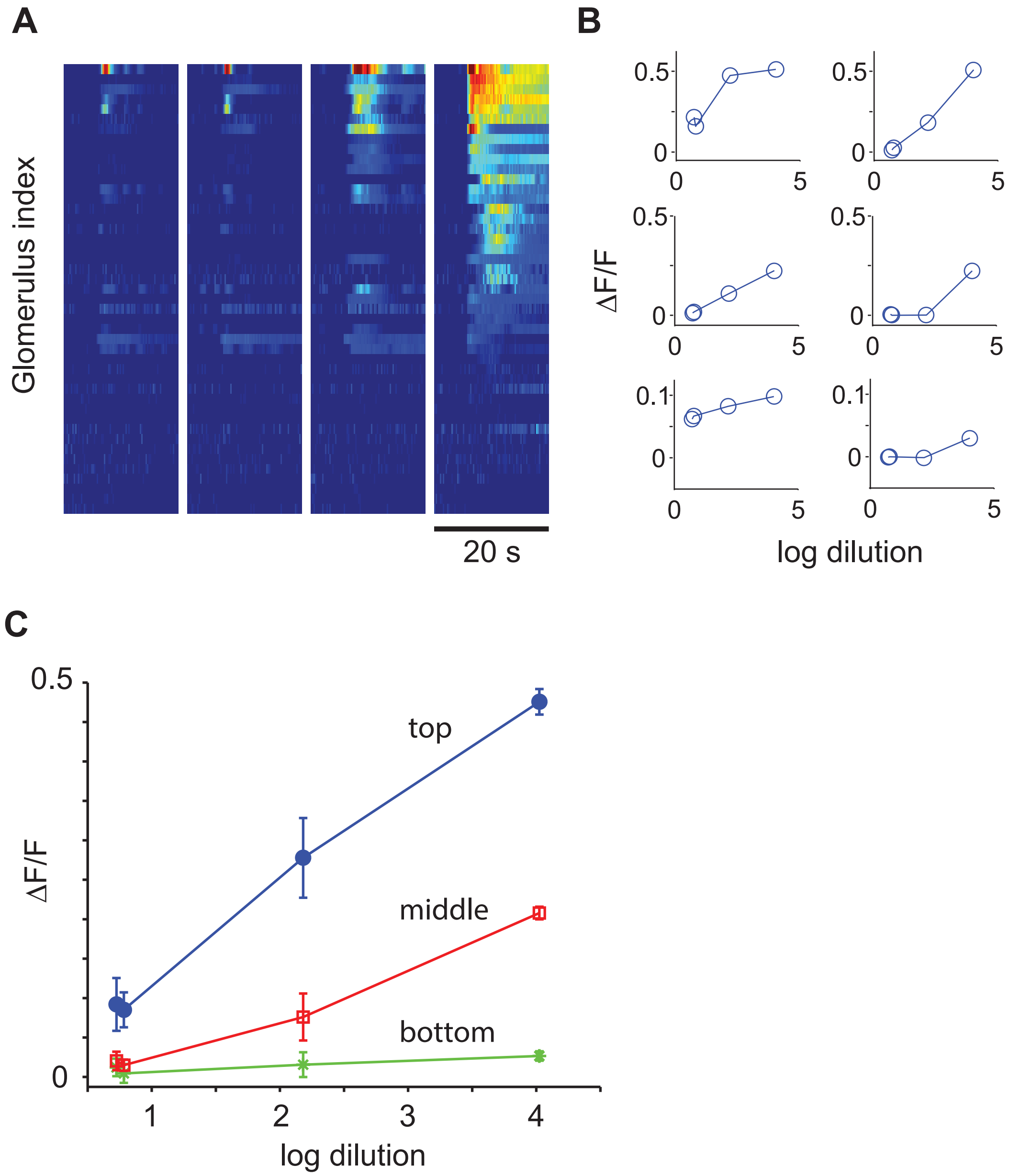

Supplementary FIgure 7

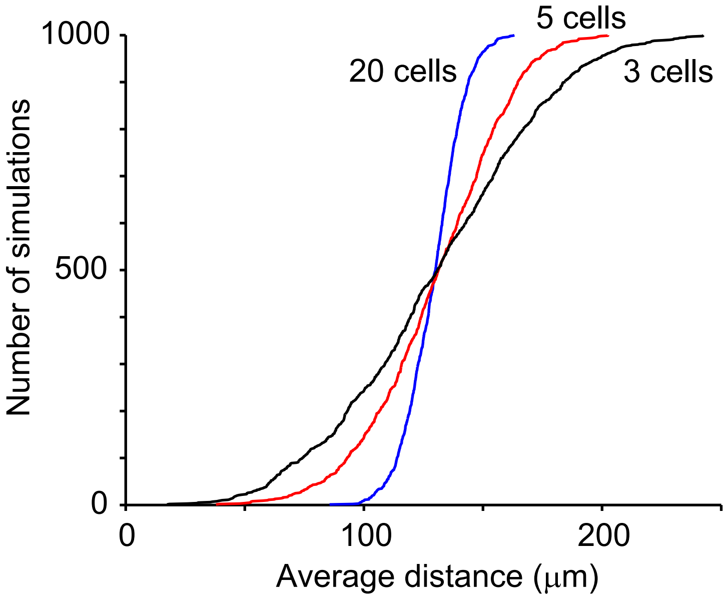

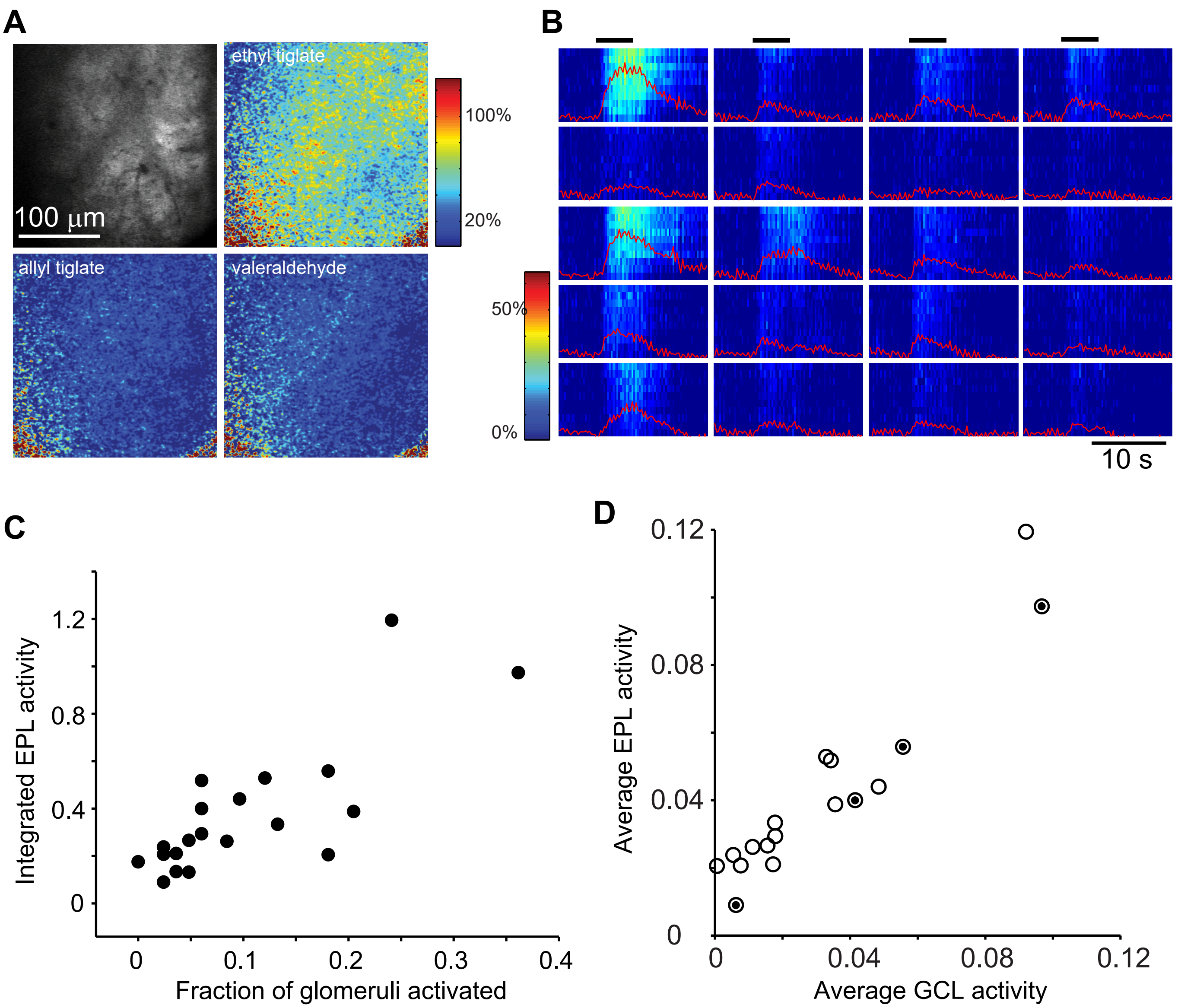
A
B
ethyl tiglate
allyl tiglate
valeraldehyde
100 μm
100%
20%
50%
0%
10 s
C
Integrated EPL activity
Fraction of glomeruli activated
0
0.4
0.8
1.2
0.1
0.2
0.3
0.4
D
Average EPL activity
Average GCL activity
0.04
0.08
0.12
